# Advances of CD19-directed chimeric antigen receptor-modified T cells in refractory/relapsed acute lymphoblastic leukemia

**DOI:** 10.1186/s40164-017-0070-9

**Published:** 2017-04-14

**Authors:** Guoqing Wei, Lijuan Ding, Jiasheng Wang, Yongxian Hu, He Huang

**Affiliations:** 1 Bone Marrow Transplantation Center, The First Affiliated Hospital, School of Medicine, Zhejiang University, Hangzhou, Zhejiang China; 2 School of Medicine, Zhejiang University, Hangzhou, Zhejiang China

**Keywords:** Acute lymphoblastic leukemia (ALL), CD19, Chimeric antigen receptor-modified T cell (CAR-T), Conditioning regimen, Complication, Cytokine release syndrome (CRS), Relapse

## Abstract

Refractory/relapsed B-cell acute lymphoblastic leukemia remains to be a significant cause of cancer-associated morbidity and mortality for children and adults. Developing novel and effective molecular-targeted approaches is thus a major priority. Chimeric antigen receptor-modified T cell (CAR-T) therapy, as one of the most promising targeted immunotherapies, has drawn extensive attention and resulted in multiple applications. According to published studies, CD19-directed CAR-T cells (CD19 CAR-T) can reach a complete remission rate of 94% in both children and adults with refractory/relapsed ALL, much higher than that of chemotherapy. However, the encouraging outcomes are often associated with complications such as cytokine release syndrome (CRS), serious neurotoxicity, and on-target off-tumor effect, which seriously impeded further clinical application of CAR-T cells. Moreover, CAR-T therapy is typically associated with high relapse rate. This article briefly reviews the manufacture technologies, the conditioning regimens, the cell infusion doses, as well as the prevention and treatment strategies of complications for CAR-T cell therapy.

## Background

The prognosis of patients with relapsed or refractory relapsed acute lymphoblastic leukemia (ALL) is generally poor. Retrospective studies have shown that the survival rate in those who relapsed depends on a variety of risk factors. The 5-year survival rate is particularly poor in those who relapsed early, estimating (21.0 ± 1.8)% in children, and less than 7% in adults [1, 2]. Although great progresses have been made in chemotherapy regimens, targeted therapies and hematopoietic stem cell transplantation, the prognosis of refractory/relapsed ALL has not been fundamentally improved.

Chimeric antigen receptor-modified T cell (CAR-T) therapy is considered one of the most promising adoptive immunotherapies at present. Chimeric antigen receptors (CARs), composed primarily of an antigen-binding domain and an intracellular T cell activation domain, redirect the modified T cells to specifically identify and eradicate malignant cells independent of MHC recognition. Although CARs have been generated against a large number of cell surface molecules such as CEQ, mesothelin and HER2, the most promising clinical outcomes were reported in B-cell leukemia and lymphoma patients treated with CD19 targeted CAR-T cells. In this review, we will briefly discuss the manufacture technologies of CAR-T cells, as well as the clinical regimens. Although CD19-directed CAR-T cells (CD19 CAR-T) have made significant progresses in the treatment of refractory/relapsed ALL, complications still hinder further clinical application. We will subsequently discuss the prevention and management of complications.

## Manufacture technologies

**Fig. 1:**
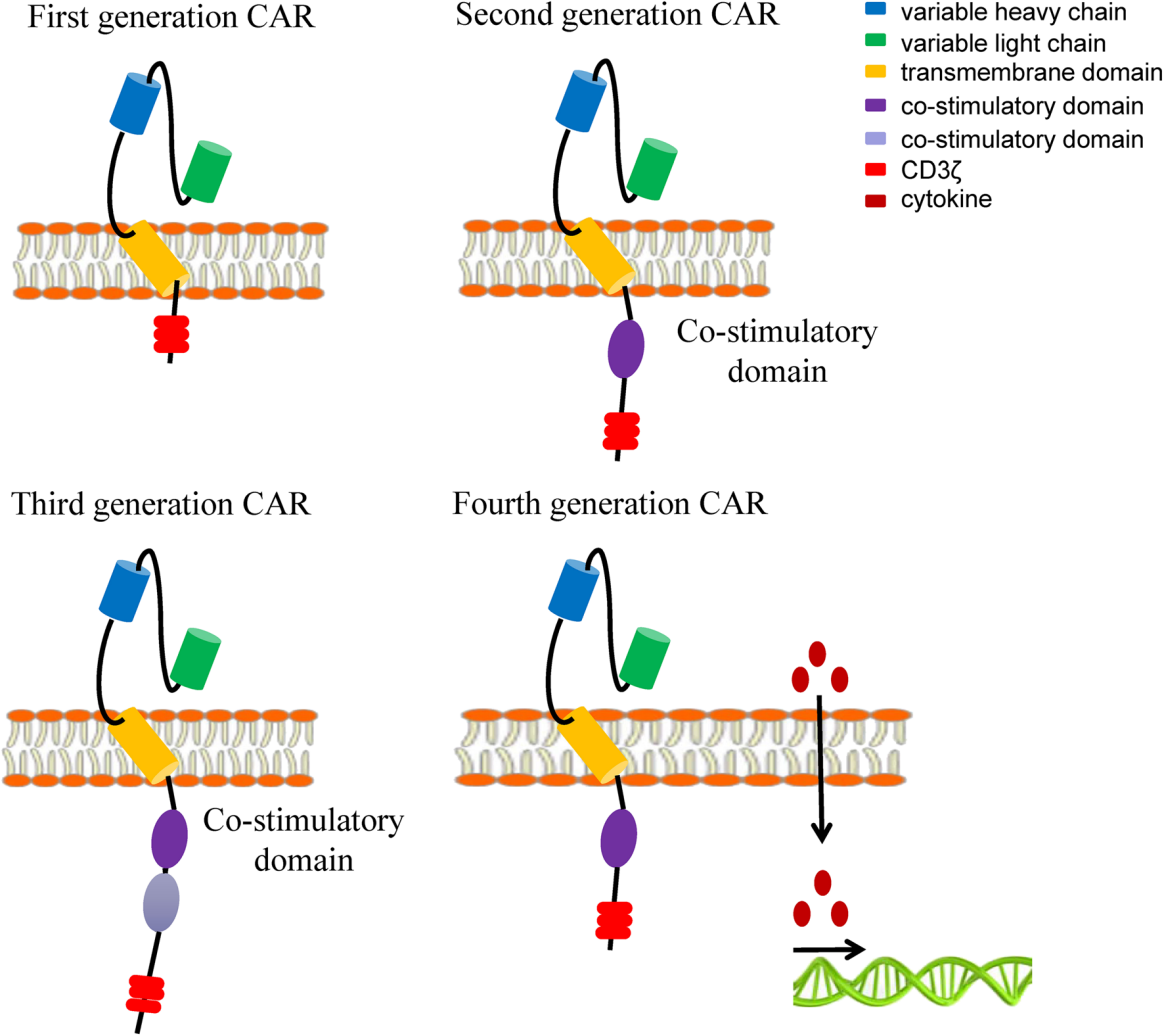
Schematic of the four-generation CARs

Chimeric antigen receptors (CARs) are synthetic receptors comprised of three key components (Fig. 1):

(1) an extracellular antigen-binding domain derived from a monoclonal antibody single chain variable fragment (scFv); (2) a transmembrane linking domain derived from CD3, CD4, CD8 or CD28; (3) an intracellular signal domain consists of CD3ζ with or without costimulatory molecules [3]. DNA constructs encoding such CARs could be stablely incorporated into human T cells via lentiviral or gamma-retroviral transductions, electroporation, as well as transposon.

### Costimulatory molecules

Most first generation CARs utilized CD3ζ as the only stimulatory molecule to activate T cells, which ultimately revealed defects of weak proliferation ability, poor anti-tumor effect, and short survival of T cells [4]. The second and third generation CARs introduced one or two costimulatory signal domains, which significantly enhanced the expansion, persistence and potency of CAR-T cells. Costimulatory molecules can be CD28, 4-1BB, CD22, CD27, OX40, or ICOS, among which CD28 and 4-1BB are currently the most widely used. Studies showed that CD28 endued CAR-T cells with stronger killing ability, while 4-1BB granted longer persistence in vivo. Some researchers have attempted to integrate CD28 and 4-1BB into one CAR molecule. Wang et al. found in pre-clinical trials that such third generation CAR-T cells showed stronger proliferating and cytokine releasing activities [5]. Different functions of costimulatory molecules could be complement, suggesting the potential value of third generation CARs for clinical application. However, Hombach et al. discovered that third generation CAR-CIK (cytokine induced killer cells) integrated with CD28 and OX40 was inclined to trigger activation-induced cell apoptosis (AICD), which considerably reduced cell survival time and tumor-killing effect [6]. The validity and safety of third generation CAR-T cells need to be further verified. At present, the fourth generation CAR-T cells constructed by vectors encoding CAR and/or CAR responsive promoters are able to produce an activation signal under the effect of cytokines encoded by CAR and to enhance the anti-tumor effect [7]. With the rapid development of CRISPR/Cas9 and other gene editing technologies, the manufacture of CAR-T cells is becoming increasingly ameliorated.

### Transfection schemes

The most efficient and widely used method to genetically engineer T cells is by viral vector transduction, using either gamma-retroviral vector or lentiviral vector [8]. The advantage of gamma-retroviral vector is the simplified processing, as well as the considerable efficiency [9]. However, gamma-retroviral vector could only be transfected into mitotic phase cells, and there have been several reports of insertional mutagenesis [10]. Lentiviral vector from human immunodeficiency virus-1 (HIV-1) can be transfected into non-dividing cells. It can carry larger objective gene fragments and cause milder immune response [11]. Therefore, CAR-T cells are currently transfected by lentiviral vectors in most cases. There are also reports of electroporation [12], but its clinical application is limited because it may take several months to achieve adequate CAR expression levels, while retroviral vector transduction takes only a month. In 1997, Ivics et al. reconstructed “Sleeping Beauty” (SB) transposon system using bioinformatic methods [13]. As the first synthetic DNA transposon, it is asserted that SB transposon can be inserted into genome randomly and ensures long-term stable expression of objective genes [14]. Huang et al. reported for the first time using SB transposon technology to manufacture CD19 CAR-T cells, and confirmed the specific killing ability targeting CD19-positive leukemia and lymphoma cells [15].

### Effector cell screening

The ideal effector cell type for CAR technology should have the following characteristics: (1) amplification can be achieved to meet the requirements of adoptive infusion both in vivo and in vitro; (2) sufficient tumor killing ability; (3) ability to mobilize to the tumor site; (4) predictable and manageable side effects [16]. As the major component of cellular immunity, T lymphocytes satisfy all the above characteristics, and thus are most widely used as effector cells for CAR technology [17]. CAR-T cells specifically recognize tumor cell surface antigen through antigen–antibody reaction and are independent of major histocompatibility complex (MHC), therefore avoiding tumor’s immune escape by down-regulation of MHC. Turtle et al. found that CAR-T cells manufactured from central memory T cells or initial T cells had stronger tumor-killing ability than those from effector memory cells [18]. CAR-T cells from different T lymphocyte subsets will be a hotspot in future research.

## Clinical application of CD19 CAR-T cells

Clinical application of CD19 CAR-T cells was first pursued in the phase I clinical trial of MSKCC, in succession followed by NCI and UPENN [19–21]. The CR rate for refractory/relapsed ALL were 88%, 67% and 90%, respectively, much higher than traditional chemotherapy. Since then, numerous studies have been carried out across the globe with similar encouraging results. Our team recently reported a CD19 CAR-T phase II clinical trial with CR rate of 94.7% in 1 month [22]. Despite the promising results, the conditioning regimen and the infusion dose differ among these studies. Joint efforts are urgently needed to form a standard protocol.

### Conditioning regimen

The conditioning regimen is among the most disputable topics in CAR-T cell therapy. Currently used regimens include cyclophosphamide, bendamustine, combination of fludarabine and cyclophosphamide (FC regimen), and combination of pentostatin and cyclophosphamide [23]. It must be admitted that we cannot decide which is the best conditioning regimen due to the limited cases. However, previous studies have shown that conditioning therapy could significantly improve the therapeutic effect. A meta-analysis by Zhang et al. showed that the 6-month progression free survival (PFS) was 94.6% for patients administrated with lymphodepletion regimen prior to cell infusion, significantly higher than 54.5% in patients without lymphodepletion (P < 0.001) [24]. Turtle et al. discovered a significantly increased duration of CAR-T cells in vivo and disease-free survival (DFS) in patients received FC conditioning regimen [18]. In conclusion, conditioning therapy can reduce tumor burden and complications such as CRS. For these reasons, developing an appropriate conditioning regimen will be helpful in improving the safety and effectiveness of CAR-T cell therapy.

### Cell infusion dose

Zhao et al. found in leukemia mouse models that CR could not be achieved when infused with less than 4 × 10^5^ CD19 CAR-T cells [25], suggesting a minimal threshold for CAR-T cell infusion. The meta-analysis by Zhang et al. showed a 6-month PFS of 94.4% in patients infused more than 10^8^ CAR-T cells, significantly higher than 58.6% in patients infused less than 10^8^ CAR-T cells (P < 0.001) [24]. On the other hand, higher infusion doses are associated with more side effects. Turtle et al. in their clinical study set a dose gradient of 2 × 10^5^/kg, 2 × 10^6^/kg and 2 × 10^7^/kg CART cells. Unfortunately, the patient who received the cell dose of 2 × 10^7^/kg died of serious CRS, while other dose groups were complicated by reversible CRS and neurotoxicity [26]. In another case report, Morgan et al. described a patient died of severe CRS within a few minutes after infusion of 10^10^ CAR-T cells [27]. The two aforementioned cases suggested that larger doses of CAR-T cells could be associated with more severe CRS, leading to significant mortality. How to optimize infusion cell dose without compromising antitumor effect or causing severe CRS requires further study.

## Complications

Common side effects of CAR-T cell therapy include chills, fever, hypotension, and leukopenia. These complications can be easily alleviated with supportive care. Nevertheless, more serious complications such as cytokine release syndrome (CRS), serious neurotoxicity (SNT), on-target off-tumor effect and relapse are more difficult to manage.

### Cytokine release syndrome (CRS)

In 1990, Chatenoud group reported the first case of CRS caused by biological products when they used anti-CD3 monoclonal antibody in patients post solid organ transplantation [28]. Outside the realm of biological products, CRS is also common and could occur in conditions like severe infection, hemophagocytic syndrome (HLH), or graft versus host disease (GvHD) [29–31]. It is generally accepted that CRS is an immune-mediated disorder. Activation of large number of T cells leads to excessive secretion of inflammatory cytokines and the recruitment and activation of other immune cells [32]. The major cytokines up-regulated during CRS are IFN-γ, IL-6, IL-10, TNF-α and IL-2 [33]. Excessive cytokines can cause systemic inflammatory responses, leading to visceral and vascular endothelial injury, heart failure, pulmonary edema, respiratory distress, microvascular leakage, among other serious complications.

Clinical manifestations and severity associated with CRS vary greatly, while fever is almost always seen in patients with CRS. Other common manifestations include headache, nausea, asthenia, hypotension, and elevations in serum aminotransferases and bilirubin. A CD19 CAR-T clinical research of Lee et al. showed that the incidence rate of CRS was 76.2%, among which grade 1–4 CRS accounted for 38.1%, 9.5%, 14.3%, and 14.3%, respectively [21]. The research also proposed a CRS grading system and the corresponding treatments based on their clinical experience. They believed that grade 3–4 CRS was the indication for using glucocorticoids and/or Tocilizumab (anti-IL-6R monoclonal antibody) [34]. It has been proved that glucocorticoids and Tocilizumab show good clinical efficacy in grade 3–4 CRS [35]. However, the effect of glucocorticoids is a double-edged sword in severe CRS, as it may jeopardize CAR-T cell function. Moreover, inappropriate use of glucocorticoids is associated with early relapse of primary disease [36].

In order to achieve early recognition and accurate diagnosis of CRS, Teachey’ team carried out a research for novel diagnostic markers of CRS [33]. Their study showed that a cytokine spectrum of IFN-γ, IL-6, IL-8, sIL-2Rα, sgp130, sIL-6R, MCP1, MIP1-α, MIP1-β and GM-CSF could be used as a predictive model for severe CRS; elevation of IFN-γ and sgp130 is strongly associated with grade 4–5 CRS. Sometimes, however, CRS can still be difficult to differentiate from infection. Searching for novel CRS markers of high sensitivity and specificity can further reduce the incidence of CRS and decrease CRS-related mortality.

### Serious neurotoxicity (SNT)

SNT can manifest as central and/or peripheral nervous system symptoms like headache, vomiting, visual changes, tremor, aphasia, hemiplegia, lethargy, seizure and myoclonus. An interesting finding is that SNT is often accompanied by CRS, and will improve when CRS improves. Most researchers agree that cytokines of CRS are the major causes of SNT, but there is no clinical proof of that. Our team first reported a case of cerebral CRS [37], finding that INF-γ, IL-6 and IL-10 in patient’ s plasma reached the level of grade 2 CRS, while the level of INF-γ, IL-6 in cerebrospinal fluid (CSF) is dozens of times of that in peripheral blood. In addition, qPCR analysis for CAR construct showed significantly higher DNA copies in CSF than that in peripheral blood. This patient achieved CR of cerebral CRS and primary disease through reducing intracranial pressure and glucocorticoids. Most centers have reported SNT to be self-limited and patients recover without long-term neurologic deficits. Supportive treatment is sufficient for mild SNT, while severe symptoms can be treated with appropriate administration of glucocorticoids. Tocilizumab is not recommended because it cannot pass the blood–brain barrier.

### On-target off-tumor effects

On-target off-tumor effects are autoimmune toxicities due to CAR-T cells attacking normal tissues outside of tumor when tumor associated antigens are expressed on non-malignant tissues. CD19 is a specific antigen of B cell lineage, which is expressed on the surface of malignant B cells as well as normal B lymphocytes. Therefore, B cell aplasia is one of the complications of CD19 CAR-T cell therapy. As the main function of B lymphocytes is to produce immunoglobulins (Ig), intravenous Ig (IVIG) infusion is helpful in B cell aplasia. In a study done by Porter et al. all patients who responded to CD19 CAR-T cell therapy developed B cell aplasia and required IVIG on a periodic basis [38]. As a matter of fact, B cell aplasia could last for up to 4 years in certain individuals. To reduce the risk of on-target off-tumor effects, the targeted antigens should be selected carefully. Wang et al. has come up with several ways to enhance selectivity of CAR, which include selecting safer antigen, combinatorial antigen targeting, tuning the sensitivity of CAR, and using the scFv derived from pro-antibody [39]. Besides, dose-escalation strategy and introducing suicide genes are all potential solutions.

### Relapse

Relapse still remains to be the major concern for CD19 CAR-T cell therapy despite promising initial CR rate. One of the common causes of relapse is CD19-negative B-ALL progression. Ruella et al. reported it consumed about 30% of all relapsed adults, while Sotillo et al. reported the rate to be (10–20)% in children [40, 41]. The mechanisms of CD19 antigen absence may include: (1) CD19 gene deletion or mutation; (2) CD19 gene transcription factor defects; (3) transformation of leukemic cell type, e.g. from lymphoid to myeloid; (4) CD19-negative clonal evolution. Grupp et al. reported six cases of CD19-positive relapse where CAR-T cells could not be detected in peripheral blood before relapse [20]. Therefore, it may be safely said that early relapse is related to the short duration of CAR-T cells. Besides, abnormal function of CAR-T cells induced by microenvironment or other factors may also be one of the mechanisms for CD19-positive relapse.

Researchers are trying various strategies to tackle the problem of relapse. Novel anti-tumor targets searching, allogeneic hematopoietic stem cell transplantation (allo-HSCT) bridged with CAR-T therapy, as well as second transfusion of CAR-T cells are currently on trial. The effects of these strategies still need further verification.

## Expectation

The 58th ASH annual meeting illustrated the eminent work that researchers had been doing in CAR-T cell therapy. Shah' team developed new CAR-T cells targeting CD22, which showed encouraging results in B-cell precursor acute lymphoblastic leukemia [42], suggesting that CD22 CAR-T cells may be a joint or remedial therapy for CD19-negative ALL and CD19-negative relapses. CD38 and CD123 may also be two important targets for refractory/relapsed ALL, as the former could reduce on-target off-tumor effects and the latter shows a strong anti-tumor effect on CD19-negative ALL mice in preclinical trials [41, 43]. Avanzi et al. demonstrated that CD19 CAR-T cells that constitutively secrete IL-18 significantly increased serum cytokine secretion, enhanced CAR-T cell persistence, induced long-term B cell aplasia and improved mouse survival, even without any prior preconditioning [44]. In addition, multiple centers are trying to develop new types of CAR-T cells, including universal CAR-T cells and hematopoietic stem cell derived CAR-T cells. De Oliveira et al. has confirmed the feasibility of incorporating CARs into hematopoietic stem cells. These modified stem cells can persist and differentiate into multiple branches of specific immune cells harboring CAR, thus effectively reducing relapses [45].

Moreover, combination therapies using CAR-T cells and other medications such as bortezomib, lenalidomide, tyrosine kinase inhibitors, immune checkpoint antibodies, and cytokines may show interesting results. One study found that lenalidomide has synergistic anti-tumor effects with CD19 CAR-T cells and CD20 CAR-T cells in treating B-Non Hodgkin’s lymphoma (B-NHL) [46].

We expect the safety and effectiveness of CD19 CAR-T cells to be further improved in the future. Through optimal designs of CAR-T cells, establishing standard protocol for conditioning and infusion, better control of complications, and combination with other therapeutic options, CD19 CAR-T cells will provide a new or the ultimate solution for the treatment of refractory/relapsed ALL.

## Abbreviations

ALL: acute lymphocytic leukemia
CAR-T: chimeric antigen receptor-modified T cell
CD19 CAR-T: CD19-directed CAR-T cell
CR: complete remission
CRS: cytokine release syndrome
CSF: cerebrospinal fluid
SNT: serious neurotoxicity
